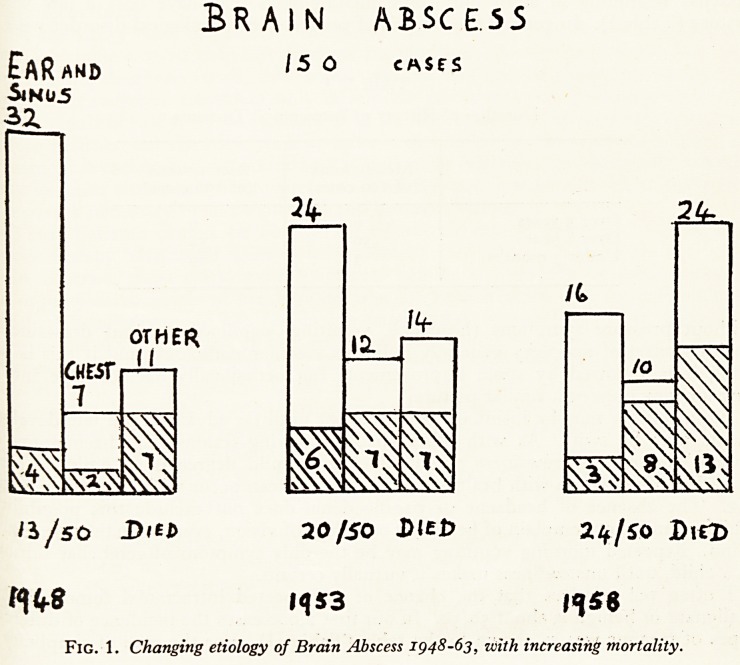# Familiar Neurosurgical Problems
*A paper given to the Bristol Medico-Chirurgical Society, 10th March 1965.


**Published:** 1965-07

**Authors:** Douglas G. Phillips

**Affiliations:** Neurosurgeon, Frenchay Hospital, Bristol


### FAMILIAR NEUROSURGICAL PROBLEMS*

BY

DOUGLAS G. PHILLIPS

*Neurosurgeon, Frenchay Hospital, Bristol*

The conditions with which I am concerned are all examples of essentially the same problem, expanding space-occupying lesions, either intracranial or spinal. These lesions account for somewhat less than half of in-patient admissions to the Neurosurgical Unit. Head injuries of all types, and intracranial tumours each number under 200 cases, out of a total admission rate of 1,000 a year.

My remarks will deal with post-traumatic intracranial haematoma, brain abscess, brain tumour, and comparable spinal conditions, in that order. Clinical differences between these conditions are in large part due to differences in the rate of growth of the expanding lesion compressing the brain or cord.

### INTRACRANIAL HAEMATOMA AFTER HEAD INJURY

Mr. George Alexander spoke to this Society in 1957 about displacements of the brain, and brainstem compression, with haematoma following head injury. Admission of cases of head injury to the Neurosurgical Unit has been selective, and concerned very largely with urgent surgery for acute intracranial haematoma. This may be the classical extradural haematoma from a middle meningeal vessel, or it may be subdural, with or without brain laceration; it may be entirely intracerebral, or a combination of these conditions. It is not possible to distinguish between them on clinical grounds.

The classical picture of a "lucid interval" after head injury, with headache, increasing impairment of consciousness, hemiparesis on the side opposite to injury, and dilating pupil on the injured side, is well known. It is dangerous to await the development of a full-blown classical syndrome. We must consider for admission without delay any patient believed to be deteriorating after head injury, even if but slightly. Many patients are urgently transferred because of suspected slight increase of drowsiness, or diminished responsiveness without any "lucid" or even "semi-lucid" interval.

Five-eighths of these acute cases were admitted with twenty-four hours of injury during the period of a recent survey (Phillips and Azariah, 1965). Haematomas were found outside and inside the dura in equal numbers. Mortality was as low as any recorded elsewhere, being 15·6 per cent in uncomplicated extradural haematoma. As might be expected it was heavier with bleeding under the dura, where actual brain laceration was present in many cases. Even then just over half survived. Nearly all patients with extradural haematoma can be expected to make an excellent recovery—after all this condition does not itself damage the brain, provided it is treated promptly, and before fatal brain-stem compression has occurred.

More surprising was the high proportion of good recoveries in cases of intradural haemorrhage where actual brain laceration may have been present with, incidentally, an increased liability to epilepsy.

The differing prognosis in these conditions is reflected in a study of mortality of patients in whom one or both pupils had become dilated and fixed either at the referring hospital or by the time of arrival at the Neurosurgical Centre. Over half such patients survived when extradural haematoma was found at operation, but almost none when the haematoma was situated more deeply.

I am not going to discuss in detail the subacute and chronic subdural haematomas. They may present with fluctuating and increasing headache, mental confusion and/or

* A paper given to the Bristol Medico-Chirurgical Society, 10th March 1965.

drowsiness a week or many months after a head injury, which may have been very minor. Of 96 patients 42 had no history of injury. Of 85 survivors, 77 were able to return to work.

### BRAIN ABSCESS

Brain abscess generally takes a little longer to develop than an acute haematoma, but it is as dangerous. If investigation and treatment of a suspected case are delayed overnight, death due to brain herniation may be sudden, without warning. The symptoms and signs are in general the same as those of any intracranial space-occupying lesion, headache, vomiting and papilloedema, and stupor, and/or focal neurological disorders, e.g. paralysis or speech disorder. Pyrexia on the other hand is characteristic of meningitis, not of brain abscess.

Stupor, as with head injury, is particularly important and may be a distinction from plain meningitis with which delirium is more usual. One should not wait for, or expect, a full picture in every case. An old axiom was that while persisting earache with chronic otitis media denoted mastoiditis requiring operation, persistent headache indicated that already there was an intracranial complication. Papilloedema is not invariable.

Formerly, most brain abscesses were associated with nasal sinusitis, or middle ear infection. This helped in diagnosis and most brain abscesses were adjacent to the focus, and solitary. The picture has changed over the past 15 years. Abscesses of otitic and sinus origin are now in the minority (Fig. 1). Most are blood-borne, some

FIG. 1. *Changing etiology of Brain Abscess 1948-63, with increasing mortality.*

from the chest, but more from elsewhere in the body. In one in six of our last 100 cases, the origin of infection remained unknown. Because of this, while the mortality of abscesses resulting from ear and sinus infection has remained low (20 per cent) the overall mortality has increased. Organisms in the lesions are a very mixed bag, with comparable numbers of coagulase positive staphylococci, non-haemolytic streptococci, anaerobic streptococci and "various" (*Streptococcus faecalis*, *Proteus*, *Ps. pyocyanea*, *B.coli* etc.). In 100 cases there were only 3 cases of pneumococcal abscesses, and 28 cases in which no organism was identified. Mortality in these last was heavy (15 deaths). Other features associated with heavy mortality were multiplicity of abscesses (18/23 deaths), associated meningitis and/or ventriculitis (19/32 deaths), and in particular the presence of deep coma on admission (16/22 deaths). If, however, an abscess does not develop such complications, the prognosis is relatively good. Of 56 survivors, 44 returned to work, even though 19 are known to have developed epilepsy. This occurs, as with brain wounds, in about 45 per cent of cases, regardless of method of treatment.

### INTRACRANIAL TUMOUR

Intracranial tumour is not so rare as was once supposed. A good many cases in the past were diagnosed as degenerative vascular disorders. The clinical presentation varies according to the situation and rate of growth of the tumour. Generally speaking the more malignant types have a short history and *vice versa*, but this cannot be relied on. Some gliomas may have a history of many years, often with isolated attacks of epilepsy beginning in adult life; some meningiomas may have only a few weeks history (Table I). In general the story is of progressive neurological disorder with or

TABLE I

Duration of History of Intracranial Tumours

| | Meningioma (of 100 cases) | Astrocytoma (of 100 cases) |
|---|---|---|
| Over 5 years | 9 | 9 |
| Over 1 year | 50 | 54 |
| Under 3 months | 3 | 29 |

without pressure symptoms (headache, vomiting, papilloedema and drowsiness). Sudden onset of apoplexy generally indicates vascular pathology, and if this is not fatal, it is followed by some improvement; but occasionally haemorrhage into a tumour may present a similar picture.

Brain tumour may be insidious or even silent until far advanced but late developments may be swift. As with other space-occupying lesions (e.g. haematoma or abscess) increasing drowsiness or stupor of even mild degree is an ominous sign. Fatal brain herniation with brain-stem compression can occur without further warning. The absence of headache or papilloedema does not exclude this possibility. Children may not complain of headache or of loss of vision, even when they are nearly blind. Repeated morning vomiting may be the only symptom of cerebellar tumour in a child, until unsteadiness makes it virtually certain.

I often tell relatives that the chance of a suspected intracranial tumour being malignant or benign is about 50/50. In our first 2,000 cases the incidence of different types of tumour was as shown in the table (Table II). For the sake of simplicity I

TABLE II

Intracranial Tumours 1948–63

| Total | 2,000 |
|---|---|
| Secondary Carcinoma | 245 |
| Glioblastoma Multiforme | 761 |
| Astrocytoma | 210 |
| Meningioma | 250 |
| Pituitary | 100 |
| Acoustic | 95 |
| Other Gliomata and various | 339 |

have named the commonest types of growth only. In general they may all present in a similar manner as "space-occupying lesions" indistinguishable on clinical grounds, though certain tumours, such as growths of the pituitary gland or acoustic nerve are more likely to present with well known syndromes.

So that the many benign treatable conditions may not be overlooked it is essential to confirm the diagnosis in all cases, usually by biopsy, though sometimes angiography or even air studies may be enough.

One need say little about secondary carcinoma, except that a very few solitary examples may benefit, even for years, from local excision, sometimes after, sometimes before, the primary source of the tumour has been identified and treated. X-ray of the chest should always be done as this may show primary tumour of the lung, or other secondaries.

Of the many cases of glioblastoma multiforme we have seen in the last 16 years, not one single proved case has survived longer than into the third year. We must confirm the diagnosis in all, but we operate on very few of these; we recommend surgical or radiation treatment only in selected favourable cases, where a temporary useful result is likely. If the tumour is in an important part of the brain with early severe neurological disorder, such as aphasia or mental confusion of some duration, treatment is useless and not a kindness to patient or relatives. In some cases radiation may be used alone, but where pressure is severe, preliminary operation and partial removal is necessary to allow survival and preserve vision.

These patients cannot be cured. All efforts by radical operation, radiation, or chemotherapy have so far failed.

In contrast, most *extra*cerebral tumours can be successfully removed. Not all. In only 27 of 100 cases of meningioma was the growth nicely over the convexity of a cerebral hemisphere. In the rest it was parasagittal, or attached to the dura at the base of the skull. In some cases it involved vital structures there, nerves, arteries or venous sinuses. Nevertheless most (65/100) could still be removed, and even when total removal was not possible, useful survival over many years could result.

Most deaths after the first five year period were attributable either to the 10 per cent of meningeal sarcoma or to extraneous causes. A very high proportion of the survivors (64/75), considering that many of these patients were past middle age, returned to work or were fit to do so. When epilepsy has been present before operation, removal of the tumour may not prevent continued liability to fits, though these are not likely to be frequent.

To offset disappointments in some cases of meningioma, the outcome of treatment in a good many of the more slowly-growing gliomas may be very good. Particularly this is so for astrocytoma of the cerebellum, occurring predominantly in children. Many of these tumours are cystic and the tumour localized to a small part of the cyst wall can be totally removed. Even where the tumour is large, solid, and sometimes involving a little of the brain stem, incomplete removal may be followed by many years (20 or more) of excellent health. The question has been raised whether most of these lesions should be classified as tumours at all. Of 100 cases of astrocytoma 28 were in the posterior fossa, and in 21 cases the patient's age was under 12 years. Nineteen of these patients remained alive and well over the next 5 to 10 years, or more. Of 72 patients with supratentorial astrocytoma a good number did well for a time. Twenty-eight returned to work and 14 remained alive and well over 5 to 10 years. Seventy-five out of 100 patients with astrocytoma had operation (including all cerebellar cases) while 42 had radiotherapy.

As others have found, acoustic tumours (which in our series were 90 per cent of intracranial neurilemmomas, the others involving the fifth or ninth nerve roots) have by no means always presented with a typical syndrome of unilateral deafness and tinnitus followed after a more or less long period by other symptoms, such as headache, trigeminal pain and sensory loss, giddiness, ataxia, nystagmus and facial palsy. Indeed in nearly half our patients other symptoms have preceded any mention of deafness or tinnitus. In thirteen cases there was no mention of tinnitus or deafness. A few patients presented simply with symptoms of raised intracranial pressure or dementia. The age spread was from 7 to 70 years, and duration of symptoms varied from 2 months to 20 years.

In many patients whose first symptoms are progressive unilateral deafness and tinnitus, study by audiometry and tests of vestibular function are of great importance if early diagnosis is to be made. Early sensory loss and weakness of face must be looked for, as well as ataxia. A high C.S.F. protein is suggestive. X-ray demonstration of an enlarged internal acoustic meatus is the most specific clue, though not always present. Careful air encephalography is of great value in doubtful cases.

Unfortunately the great majority of patients already have large tumours by the time they are diagnosed. If the tumour is really small (less than 3 cms diameter) removal is very easy and the facial nerve can be spared. With *very* small tumours the cochlear division of the eighth nerve, with hearing, may be spared (McKissock, 1961).

Of our 99 patients, 65 had a total removal; in 8 of these the facial nerve was spared. When the facial nerve has inevitably to be sacrificed with total removal of a large tumour, hypoglossal-facial anastomosis restores tone to the affected side of the face and a fair movement with speech. This is superior to accessory-facial anastomosis, or any sling procedure.

Though mortality is bound to remain substantial with large tumours, 64 of our 72 survivors returned to work or were fit to do so, for periods up to 15 years or more. For some who at first were considerably disabled, performance progressively improved over the first year or two.

The great majority of pituitary tumours are chromophobe adenomas, very frequently though not always associated with hypopituitarism. A lesser number are chromophil adenomas and are associated with acromegaly. Basophil adenomas are very rare.

The great majority of patients with pituitary tumour have visual impairment as their principal complaint. This is the main indication for operation, headache being less often important. Endocrine disturbance is not an indication for operation. Bitemporal hemianopia is commonly present in these cases—though homonymous and other partial visual field deficiencies may be found. Loss of central vision with impaired visual acuity is particularly serious. In some cases vision in one eye is totally lost, or almost totally lost before the patient complains. As with other cases of intracranial tumour, severe loss of vision is a very urgent matter. If it is far gone it may be irrecoverable, and may even progress to complete blindness after the compression of the optic nerves has been relieved. Fortunately in nearly all cases of pituitary adenoma, unless the tumour is very large, operation is followed by substantial if not complete recovery of vision.

If the growth is massive, the larger portion is outside the sella, and intimately involved with the base of the brain and other structures. This was so in nineteen of our cases. Mortality in such cases is bound to be heavy. The discovery and use of endocrine substitutes (A.C.T.H. and cortisone) has greatly added to the safety of operations for pituitary tumour.

The object of operation is to remove the main mass of the tumour pressing on the optic nerves and chiasm; active normal pituitary gland remains and functions in the sella turcica.

Radiotherapy is given alone in some early cases when impairment of vision is slight. If visual impairment is already severe, immediate post-radiation swelling may further damage remaining nerve fibres. Post-operative radiation has been given in about half our cases, as it is believed to diminish the chance of late recurrence.

Half of the ten patients dying at some time post-operatively in our series had tumours of the massive variety. Some were before the era of A.C.T.H. and cortisone. A minority of our patients (sixteen out of fifty-five where data are available) have required to be maintained on cortisone permanently.

It is important that periodic careful examination of visual fields and acuity should be done for an indefinite period, so that any recurrent loss of vision can be detected early.

Other tumours, cysts, and aneurysms about the sella and the cerebello-pontine angle may be clinically indistinguishable from pituitary and acoustic tumours.

### SPINAL TUMOURS

Turning now to *spinal* space-occupying lesions, I will not dwell on haematomas, which are rare. Abscesses are not common but we see one case of epidural abscess a year. This usually begins with pain in the back, followed by root pains and then impairment of cord functions. Immediate surgical drainage is required before compression and thrombosis in blood vessels supplying the cord causes irreparable paraplegia. This may happen in a few hours after onset of neurological disturbance so operation is very urgent in such cases.

Spinal tumours are more common. Though not so frequent as brain tumours, they may also be divided into those with good and those with bad prognosis. Again excluding secondary carcinoma, these correspond approximately to extramedullary and intramedullary tumours (Table III). The traditional textbook method of dis-

TABLE III

Spinal Tumours

100 Cases (1959–63)

| | | |
|---|---|---|
| Extramedullary .. .. .. .. | | 84 |
| Meningioma, Neurinoma .. .. | 41 | |
| Carcinoma (and Sarcoma) .. .. | 20 | |
| Others (Myeloma, Dermoids, Cysts) .. | 23 | |
| Intramedullary .. .. .. .. | | 16 |
| (Astrocytoma, Ependymoma, Cysts) | | |

tinguishing these clinically by the patterns of sensory loss is a useless and misleading exercise. It is enough to recognize that motor and sensory loss in limbs and trunk, especially but not exclusively if bilateral and progressive, may mean cord or root compression. Sphincter disturbance, which is especially serious, may present as an initial symptom and should lead to very careful examination for weakness in the lower limbs, and sensory loss in the saddle area, including the perineum.

Apart from secondaries, meningiomas and neurinomas are the largest single group of spinal tumours. Like their intracranial counterparts, they have an excellent prognosis—provided the syndrome is not allowed to progress to complete or near-complete paraplegia. When progress is rapid, it is hazardous to spend too much time eliminating the possibility of a primary growth elsewhere.

Other lesions such as cysts and dermoids also have an excellent prognosis. Some of the gliomas may benefit for a long time from decompression and partial removal.

Acute spinal compression may be caused by an intervertebral disc lesion at any level but I will consider only one variety of this condition—as a tailpiece.

### MASSIVE PROLAPSE OF LUMBAR INTERVERTEBRAL DISC WITH CAUDIA EQUINA COMPRESSION

In a recent case severe back pain for one month required repeated morphia, and at first this led to the onset of weakness of the legs being overlooked. Examination showed very limited analgesia about the anus and over the coccyx. Fortunately there was little sphincter disturbance. Myelography confirmed a spinal block. At operation the prolapsed disc was found in the extradural space dorsal to the theca at the second lumbar level, having migrated up there from the narrowed L3–4 disc interspace. Relief of pain was immediate, and motor recovery began a few days later.

We see two patients a year with prolapsed disc and cauda equina compression—in one case this followed a manipulation.

Some have not been so fortunate as the one described. One patient had pain in his back and legs following strenuous digging. After four days he developed numbness in the rectum and back of the legs, which were weak. Urinary retention followed. Three days later at operation a large fragment of protruded disc was removed from the upper lumbar spinal canal. He improved slowly but four years later he still wore leg irons and a urinal at work. Sensory loss was unchanged. He was impotent. He still wears calipers 11 years after operation.

Of four patients who had no recovery, one had 11 days numbness of penis and perineum with urinary retention. He died of pyelonephritis 2 years later. Other patients have shown varying degrees of recovery. Some sensory loss tends to persist in the saddle area. Sphincter disturbance is most serious. Four patients came to operation within 12 to 24 hours after onset of sacral motor and sensory loss with urinary retention. They made good recoveries with slight residual sensory and motor deficiency, though in one patient whose operation was done within 12 hours of urinary retention, recovery took over 2 years.

Fortunately these cases are not very common in relation to spinal disc pathology in general. However, they show well that, like the grave, the spine is "a fine and narrow place". Nerve roots and cord therein embraced soon come to harm.

To conclude, I will quote from a paper on spinal abscess published 11 years ago by Hulme and Dott (1954).

"With early recognition and treatment prior to the onset of gross neurological abnormalities, satisfactory recovery can be anticipated in most cases; while delay until signs of severe cord damage become apparent, usually results in permanent crippling disability.

"The most important factor in early diagnosis is an awareness of the possibility of the condition, and an appreciation of the rapidity with which the pathological process advances to an irreversible state".

The same considerations apply to other forms of spinal compression, and with little modification to brain-stem compression by intracranial space-occupying lesions.

### REFERENCES

Alexander, G. L. (1957). *The Med. Journ. of the South West*, **72**, 91.
Hulme, A., and Dott, N. M. (1954). *Brit. Med. J.*, **1**, 64.
McKissock, W. (1961). *J. Neurol. Neurosurg. Psychiat.*, **24**, 297.
Phillips, D. G., and Azariah, R. (1965). *Brit. J. Surg.*, **52**, 218.

## Figures and Tables

**Fig. 1. f1:**